# Supplementary material for: Introducing the EMPIRE Index: A novel, value-based metric framework to measure the impact of medical publications
Source: PLoS One. 2022 Apr 4;17(4):e0265381. doi: 10.1371/journal.pone.0265381 (PMC8979442; doi:10.1371/journal.pone.0265381)
Supplement: S1 Table — Coverage is the proportion of articles with > 0 on that metric. (DOCX) [file pone.0265381.s001.docx]

**S1 Table. Summary statistics for metrics obtained via (A) Altmetric, (B) PlumX, and (C) journal-level, citation-based indices.** Coverage is the proportion of articles with > 0 on that metric.

**(A)**

| Metric | Coverage | Mean | Median | Maximum |
| --- | --- | --- | --- | --- |
| News mentions | 0.35 | 6.4 | 0 | 365 |
| Blog mentions | 0.14 | 0.4 | 0 | 27 |
| Policy mentions | 0.08 | 0.1 | 0 | 4 |
| Patent mentions | 0.01 | 0.0 | 0 | 8 |
| Twitter mentions | 0.85 | 32.6 | 5 | 1701 |
| Original tweets | 0.85 | 10.5 | 3 | 347 |
| Retweets | 0.59 | 22.2 | 1 | 1533 |
| Peer review mentions | 0.00 | 0.0 | 0 | 2 |
| Weibo mentions | 0.00 | 0.0 | 0 | 1 |
| Facebook mentions | 0.33 | 1.3 | 0 | 55 |
| Wikipedia mentions | 0.02 | 0.0 | 0 | 8 |
| Google+ mentions | 0.08 | 0.1 | 0 | 10 |
| LinkedIn mentions | 0.00 | 0.0 | 0 | 0 |
| Reddit mentions | 0.04 | 0.0 | 0 | 6 |
| Pinterest mentions | 0.00 | 0.0 | 0 | 0 |
| F1000Prime mentions | 0.07 | 0.1 | 0 | 3 |
| Q&A mentions | 0.00 | 0.0 | 0 | 1 |
| Video mentions | 0.01 | 0.0 | 0 | 4 |
| Syllabi mentions | 0.00 | 0.0 | 0 | 0 |
| Mendeley readers | 0.99 | 43.2 | 24 | 1325 |
| Dimensions citations | 0.94 | 27.3 | 9 | 2021 |

**(B)**

| Metric | Coverage | Mean | Median | Maximum |
| --- | --- | --- | --- | --- |
| Captures:Exports-Saves:EBSCO | 0.47 | 6.0 | 0 | 456 |
| Captures:Readers:Mendeley | 0.80 | 41.6 | 19 | 1286 |
| Captures:Readers:CiteULike | 0.01 | 0.0 | 0 | 3 |
| Citations:Clinical Citations:PubMed Guidelines | 0.05 | 0.1 | 0 | 3 |
| Citations:Clinical Citations:DynaMed Plus | 0.16 | 0.2 | 0 | 5 |
| Citations:Citation Indexes:Scopus | 0.92 | 24.9 | 8 | 1971 |
| Citations:Citation Indexes:PubMed | 0.23 | 3.9 | 0 | 795 |
| Citations:Citation Indexes:CrossRef | 0.73 | 9.3 | 2 | 454 |
| Social Media:Tweets:Twitter | 0.78 | 28.0 | 3 | 1892 |
| Social Media:Shares, Likes & Comments:Facebook | 0.21 | 36.5 | 0 | 39,422 |
| Mentions:References:Wikipedia | 0.03 | 0.0 | 0 | 7 |
| Mentions:Blog | 0.11 | 0.2 | 0 | 13 |
| Mentions:Comments:Reddit | 0.00 | 0.0 | 0 | 17 |
| Mentions:News Mentions:News | 0.29 | 3.1 | 0 | 261 |
| Usage:Views:Figshare | 0.01 | 0.3 | 0 | 68 |
| Usage:Abstract Views:DSpace | 0.00 | 0.5 | 0 | 315 |
| Usage:Abstract Views:SciELO | 0.00 | 0.1 | 0 | 87 |
| Usage:Abstract Views:Digital Commons | 0.02 | 0.2 | 0 | 58 |
| Usage:Abstract Views:Expert Gallery Suite | 0.00 | 0.0 | 0 | 8 |
| Usage:Abstract Views:EBSCO | 0.71 | 68.9 | 16 | 3124 |
| Usage:Clicks:Bitly | 0.13 | 7.6 | 0 | 2862 |
| Usage:Downloads:Figshare | 0.01 | 0.2 | 0 | 76 |
| Usage:Downloads:UWA Research Repository | 0.00 | 0.0 | 0 | 6 |
| Usage:Downloads:Digital Commons | 0.01 | 0.5 | 0 | 220 |
| Usage:Downloads:Expert Gallery Suite | 0.00 | 0.0 | 0 | 7 |
| Usage:Downloads:EBSCO | 0.00 | 0.0 | 0 | 1 |
| Usage:Full Text Views:PubMedCentral | 0.02 | 9.9 | 0 | 2119 |
| Usage:Full Text Views:SciELO | 0.00 | 0.8 | 0 | 1064 |
| Usage:Full Text Views:PLoS | 0.02 | 27.5 | 0 | 7710 |
| Usage:Full Text Views:EBSCO | 0.13 | 3.7 | 0 | 2663 |
| Usage:Link-outs:EBSCO | 0.56 | 7.1 | 1 | 374 |

**(C)**

| Metric | Density | Mean | Median | Maximum |
| --- | --- | --- | --- | --- |
| CiteScore | 1.00 | 5.3 | 4.03 | 19.14 |
| Journal Impact Factor | 0.91 | 14.1 | 5.231 | 79.258 |
| Scimago Journal Ranking | 0.79 | 4.6 | 2.339 | 19.476 |
