# Supplementary material for: Introducing the EMPIRE Index: A novel, value-based metric framework to measure the impact of medical publications
Source: PLoS One. 2022 Apr 4;17(4):e0265381. doi: 10.1371/journal.pone.0265381 (PMC8979442; doi:10.1371/journal.pone.0265381)
Supplement: S2 Table — Correlations > 0.5 are shown in bold. (DOCX) [file pone.0265381.s002.docx]

**S2 Table. Correlations (Spearman’s r) between investigational metrics in the sample of Phase III clinical trial publications.** Correlations > 0.5 are shown in bold.

| **Metric** | **News mentions** | **Blog mentions** | **Policy mentions** | **Patent mentions** | **Twitter mentions** | **Original tweets** | **Retweets** | **Facebook mentions** | **Wikipedia mentions** | **F1000 mentions** | **Mendeley readers** | **Dimen-sions citations** | **PubMed guidelines** |
| --- | --- | --- | --- | --- | --- | --- | --- | --- | --- | --- | --- | --- | --- |
| **News mentions** | – | **0.529** | 0.165 | 0.066 | **0.530** | **0.535** | **0.519** | 0.479 | 0.160 | 0.302 | **0.504** | **0.518** | 0.187 |
| **Blog mentions** | **0.529** | – | 0.136 | 0.062 | 0.455 | 0.461 | 0.459 | 0.476 | 0.191 | 0.327 | 0.406 | 0.373 | 0.127 |
| **Policy mentions** | 0.165 | 0.136 | – | 0.044 | 0.116 | 0.121 | 0.120 | 0.130 | 0.086 | 0.101 | 0.185 | 0.220 | 0.134 |
| **Patent mentions** | 0.066 | 0.062 | 0.044 | – | 0.048 | 0.050 | 0.045 | 0.048 | 0.073 | 0.038 | 0.073 | 0.110 | 0.098 |
| **Twitter mentions** | **0.530** | 0.455 | 0.116 | 0.048 | – | **0.958** | **0.932** | **0.555** | 0.140 | 0.270 | **0.539** | **0.521** | 0.140 |
| **Original tweets** | **0.535** | 0.461 | 0.121 | 0.050 | **0.958** | – | **0.819** | **0.566** | 0.140 | 0.266 | **0.552** | **0.522** | 0.145 |
| **Retweets** | **0.519** | 0.459 | 0.120 | 0.045 | **0.932** | **0.819** | – | **0.534** | 0.145 | 0.283 | **0.509** | **0.505** | 0.138 |
| **Facebook mentions** | 0.479 | 0.476 | 0.130 | 0.048 | **0.555** | **0.566** | **0.534** | – | 0.155 | 0.288 | 0.448 | 0.417 | 0.130 |
| **Wikipedia mentions** | 0.160 | 0.191 | 0.086 | 0.073 | 0.140 | 0.140 | 0.145 | 0.155 | – | 0.163 | 0.130 | 0.138 | 0.057 |
| **F1000Prime mentions** | 0.302 | 0.327 | 0.101 | 0.038 | 0.270 | 0.266 | 0.283 | 0.288 | 0.163 | – | 0.264 | 0.284 | 0.118 |
| **Mendeley readers** | **0.504** | 0.406 | 0.185 | 0.073 | **0.539** | **0.552** | **0.509** | 0.448 | 0.130 | 0.264 | – | **0.737** | 0.221 |
| **Dimensions citations** | **0.518** | 0.373 | 0.220 | 0.110 | **0.521** | **0.522** | **0.505** | 0.417 | 0.138 | 0.284 | **0.737** | – | 0.274 |
| **PubMed guidelines mentions** | 0.187 | 0.127 | 0.134 | 0.098 | 0.140 | 0.145 | 0.138 | 0.130 | 0.057 | 0.118 | 0.221 | 0.274 | **–** |
