# Supplementary material for: Introducing the EMPIRE Index: A novel, value-based metric framework to measure the impact of medical publications
Source: PLoS One. 2022 Apr 4;17(4):e0265381. doi: 10.1371/journal.pone.0265381 (PMC8979442; doi:10.1371/journal.pone.0265381)
Supplement: S3 Table — Highest loadings for each metric are shown in bold. (DOCX) [file pone.0265381.s003.docx]

**S3 Table. Three-factor analysis of included metrics in (A) the full sample, (B) older papers (1H), and (C) younger papers (2H).** Highest loadings for each metric are shown in bold.

**(A)**

| **Metric** | **1** | **2** | **3** |
| --- | --- | --- | --- |
| News mentions | **−0.54** | 0.19 | 0.14 |
| Blog mentions | **−0.88** | −0.02 | −0.01 |
| Policy mentions | −0.18 | **0.32** | −0.17 |
| Patent mentions | −0.12 | **0.14** | −0.14 |
| Twitter mentions | −0.02 | 0.06 | **0.84** |
| Facebook mentions | −0.43 | 0.01 | **0.43** |
| Wikipedia mentions | **−0.33** | 0.05 | −0.09 |
| F1000Prime mentions | **–0.42** | 0.11 | 0.00 |
| Mendeley readers | −0.06 | **0.65** | 0.17 |
| Dimensions citations | 0.02 | **0.96** | −0.01 |
| PubMed guidelines mentions | −0.07 | **0.40** | −0.17 |

**(B)**

| **Metric** | **1** | **2** | **3** |
| --- | --- | --- | --- |
| News mentions | −0.19 | 0.04 | **−0.58** |
| Blog mentions | 0.00 | −0.01 | **−0.84** |
| Policy mentions | **–0.29** | −0.06 | −0.19 |
| Patent mentions | **−0.13** | −0.05 | −0.09 |
| Twitter mentions | −0.08 | **0.87** | 0.00 |
| Facebook mentions | 0.09 | **0.42** | −0.48 |
| Wikipedia mentions | −0.06 | −0.06 | **−0.31** |
| F1000Prime mentions | −0.13 | −0.04 | **−0.45** |
| Mendeley readers | **−0.65** | 0.13 | −0.05 |
| Dimensions citations | **−0.93** | 0.01 | 0.00 |
| PubMed guidelines mentions | **−0.41** | −0.10 | −0.08 |

**(C)**

| **Metric** | **1** | **2** | **3** |
| --- | --- | --- | --- |
| News mentions | −0.11 | −0.25 | **0.52** |
| Blog mentions | 0.03 | 0.04 | **0.92** |
| Policy mentions | 0.16 | **−0.26** | 0.12 |
| Patent mentions | 0.11 | −0.03 | **0.15** |
| Twitter mentions | **−0.98** | 0.00 | 0.01 |
| Facebook mentions | −0.26 | −0.12 | **0.54** |
| Wikipedia mentions | 0.09 | −0.05 | **0.30** |
| F1000Prime mentions | −0.03 | −0.09 | **0.39** |
| Mendeley readers | −0.07 | **−0.71** | 0.11 |
| Dimensions citations | 0.02 | **−0.96** | −0.04 |
| PubMed guidelines mentions | 0.04 | **−0.25** | −0.03 |
