# Supplementary material for: Introducing the EMPIRE Index: A novel, value-based metric framework to measure the impact of medical publications
Source: PLoS One. 2022 Apr 4;17(4):e0265381. doi: 10.1371/journal.pone.0265381 (PMC8979442; doi:10.1371/journal.pone.0265381)
Supplement: S4 Table — Highest loadings for each metric are shown in bold. (DOCX) [file pone.0265381.s004.docx]

**S4 Table. Two-factor analysis of metrics excluding citations in policy documents, PubMed guidelines, and patents in (A) the full sample, (B) older papers (1H), and (C) younger papers (2H).** Highest loadings for each metric are shown in bold.

**(A)**

| **Metric** | **1** | **2** |
| --- | --- | --- |
| News mentions | **0.68** | −0.13 |
| Blog mentions | **0.86** | 0.09 |
| Twitter mentions | **0.65** | −0.19 |
| Facebook mentions | **0.84** | 0.02 |
| Wikipedia mentions | **0.27** | 0.01 |
| F1000Prime mentions | **0.44** | −0.05 |
| Mendeley readers | 0.14 | **−0.73** |
| Dimensions citations | −0.04 | **−0.93** |

**(B)**

| **Metric** | **1** | **2** |
| --- | --- | --- |
| News mentions | 0.15 | **0.62** |
| Blog mentions | −0.04 | **0.81** |
| Twitter mentions | 0.34 | **0.49** |
| Facebook mentions | −0.05 | **0.81** |
| Wikipedia mentions | 0.01 | **0.28** |
| F1000Prime mentions | 0.07 | **0.44** |
| Mendeley readers | **0.77** | 0.06 |
| Dimensions citations | **0.91** | −0.02 |

**(C)**

| **Metric** | **1** | **2** |
| --- | --- | --- |
| News mentions | **−0.66** | −0.18 |
| Blog mentions | **−0.91** | 0.12 |
| Twitter mentions | **−0.63** | −0.24 |
| Facebook mentions | **−0.83** | −0.04 |
| Wikipedia mentions | **−0.25** | 0.01 |
| F1000 mentions | **−0.46** | −0.02 |
| Mendeley readers | −0.17 | **−0.71** |
| Dimensions citations | 0.03 | **−0.93** |
