# Supplementary figures and images for: Introducing the EMPIRE Index: A novel, value-based metric framework to measure the impact of medical publications
Source: PLoS One. 2022 Apr 4;17(4):e0265381. doi: 10.1371/journal.pone.0265381 (PMC8979442; doi:10.1371/journal.pone.0265381)

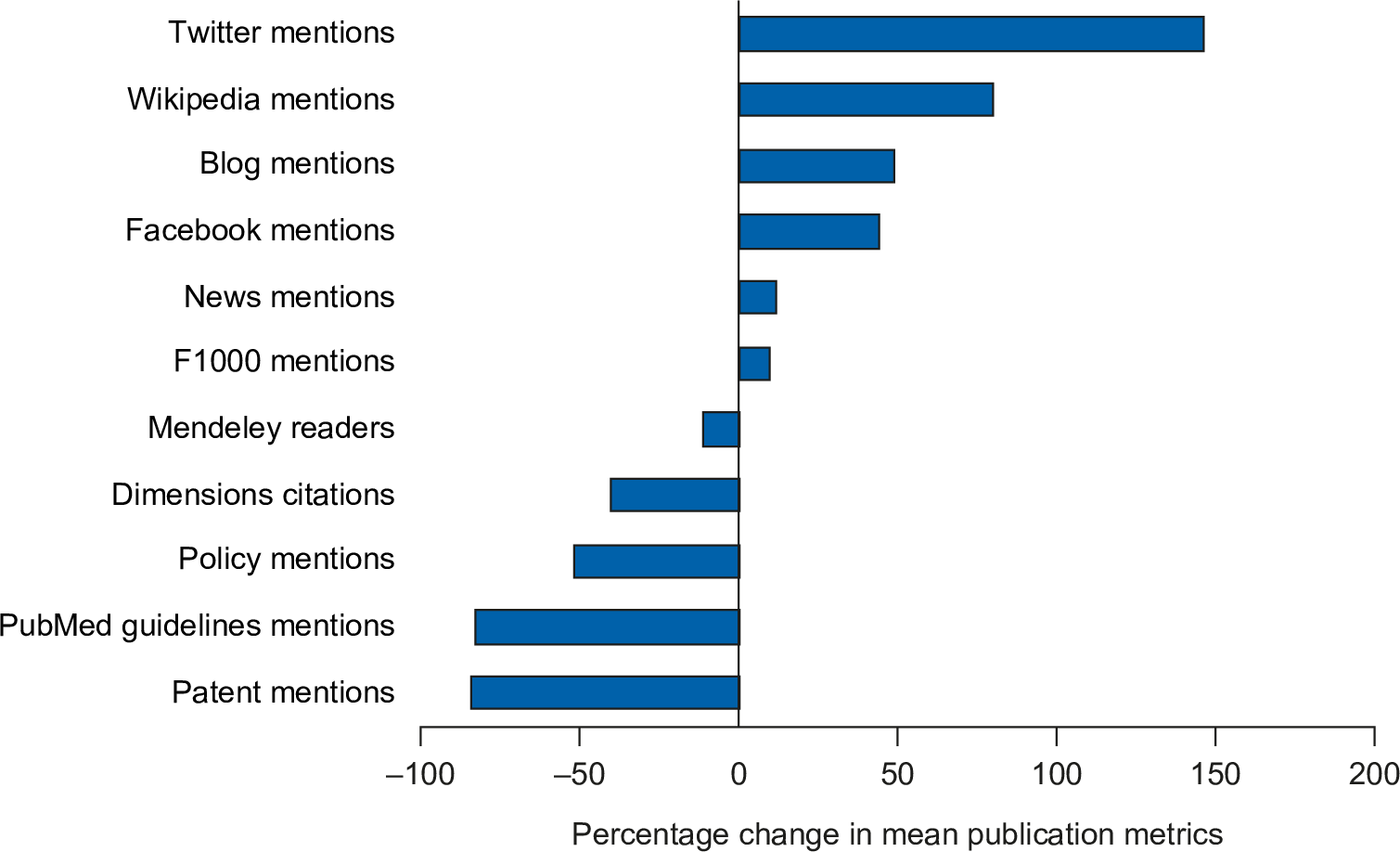

Supplement: S1 Fig — (TIF) [file pone.0265381.s005.tif]

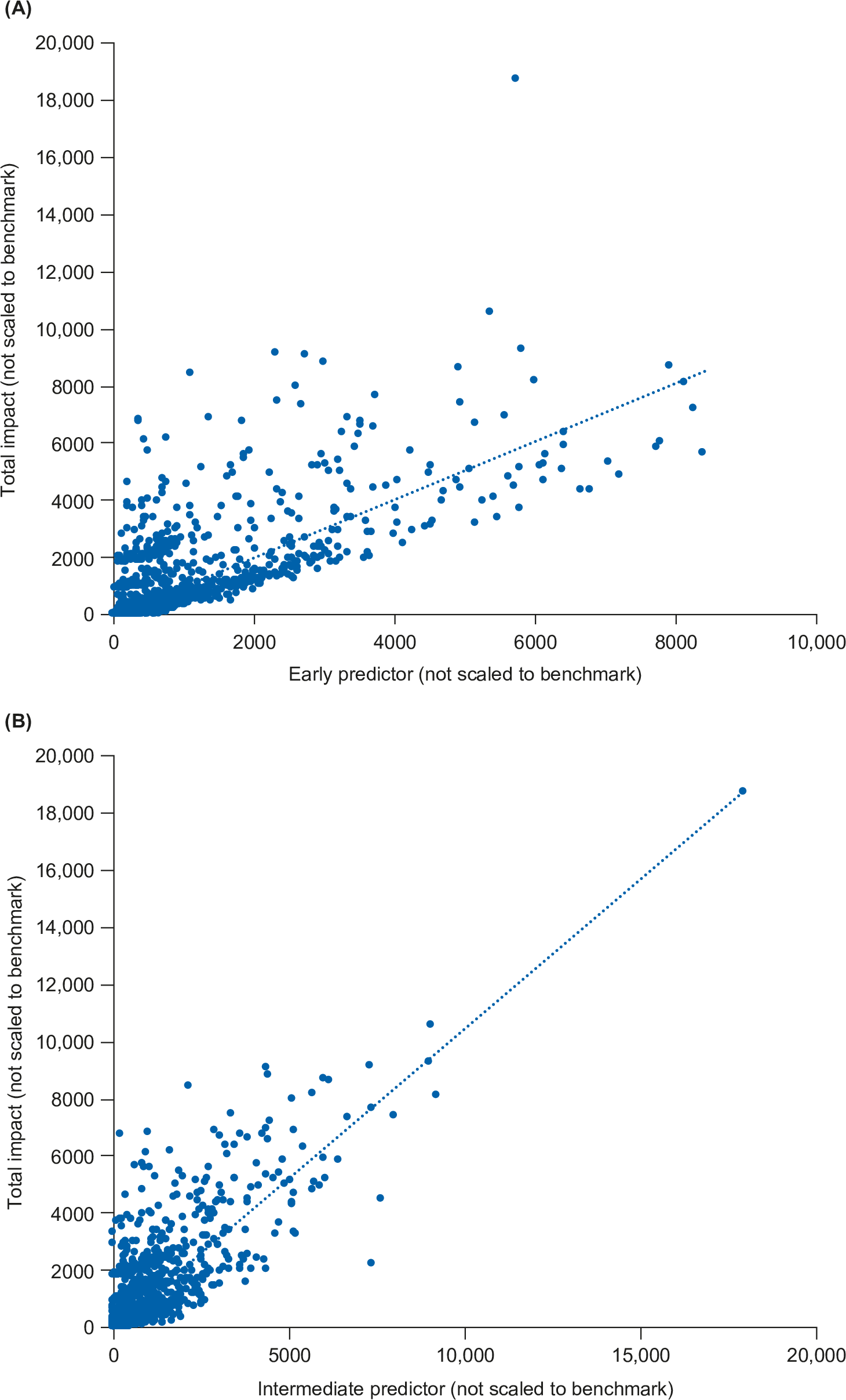

Supplement: S2 Fig — Scores shown are not adjusted to the benchmark. (TIF) [file pone.0265381.s006.tif]
